# Etiologic assessment of palatal petechiae – a case report

**DOI:** 10.3205/dgkh000523

**Published:** 2024-12-16

**Authors:** Karthik Shunmugavelu, Subhashini Paari

**Affiliations:** 1 PSP Medical College Hospital and Research Institute Tambaram Kanchipuram, Tamil Nadu, India; 2 Sree Balaji Medical College and Hospital Chennai, Tamil Nadu, India

**Keywords:** fellatio, oral lesion, palatal petechiae

## Abstract

Because oral lesions can develop from various etiological factors, it is of utmost importance to obtain a comprehensive history and oral examination. Fellatio is a commonly practiced sexual act, which may result in submucosal hemorrhage of the palate. The lesions are asymptomatic and typically appear on the soft palate. We report a case of a 57-year-old woman who presented with an incidental erythematous lesion about 3 cm with a clear center on her soft palate during her dental visit. As the patients may be unaware of the etiology of the lesions and when they hesitate to provide the details of the sexual history, it is important that the clinician, based on the patient’s clinical presentation, consider fellatio as a possible cause.

## Abstract (de)

Orale Läsionen können durch unterschiedliche ätiologische Faktoren entstehen. Daher ist es von größter Bedeutung, eine umfassende Anamnese und orale Untersuchung durchzuführen. Fellatio ist ein häufig praktizierter sexueller Akt, der zu submukösen Blutungen am Gaumen führen kann. Die Läsionen sind asymptomatisch und treten typischerweise am weichen Gaumen auf. In diesem Bericht berichten wir über eine 57-jährige Frau, die sich bei ihrem Zahnarztbesuch mit einer ca. 3 cm großen erythematösen Läsion mit klarem Zentrum am weichen Gaumen vorstellte. Da sich die Patienten der Ätiologie der Läsionen möglicherweise nicht bewusst sind und zögern, Einzelheiten zur Sexualanamnese anzugeben, ist es wichtig, dass der Arzt aufgrund der klinischen Präsentation Fellatio in die Ursachenklärung einbezieht.

## Introduction

Erythematous lesions of the oral mucosa are common and can reflect a variety of conditions, ranging from benign reactive or immunologically-mediated disorders to malignant disease [1]. A wide range of palatal lesions can be found during oral examinations, with clinical presentations such as ecchymosis, erythema, purpura and petechiae [2]. Fellatio has been reported to be popular among the young population as it is considered by them to be a safer mode of sex [3].

## Background

The penile glands contacting the palate during fellatio may create a hematoma due to blunt trauma and dilation of the blood vessels caused by the negative pressure created while sucking [4]. Palatal petechiae may develop due to negative pressure, and fellacio-associated palatal petechiae results from direct and forceful contact of the distal penis against the palate along with negative pressure, resulting in mucosal injury with rupture of submucosal vessels and hemorrhage [5].

## Case description

**Figure 1:**
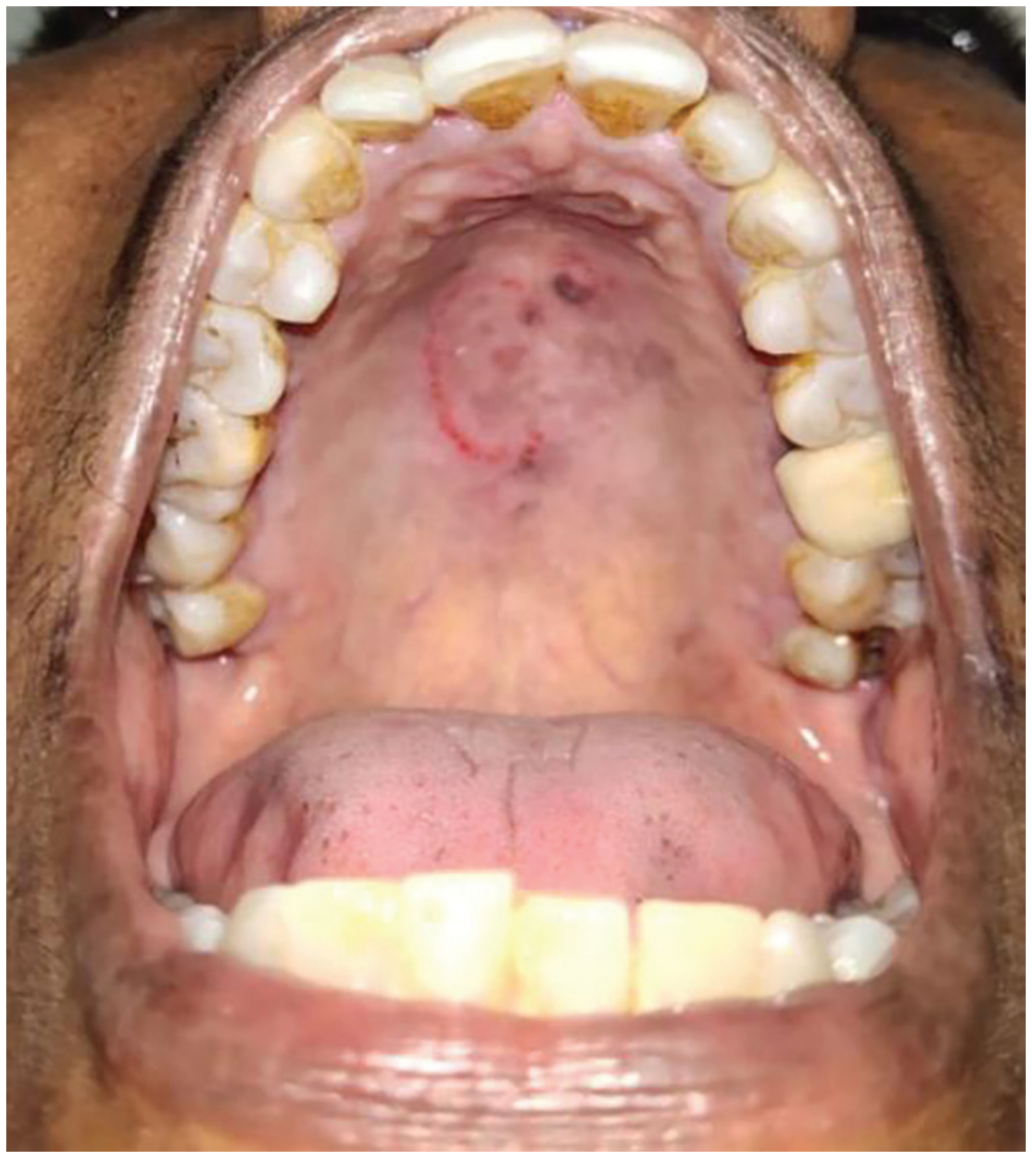
Erythematous palatal petechiae with a clear center of about 3 cm

A 57-year-old female patient visited the dental office with the chief complaint of pain in the right mandibular molar region. The pain was a dull ache and intermittent in nature. Upon intra-oral examination, caries in tooth 36 was noted. It demonstrated mild tenderness on percussion. A radiographic image revealed pulpal involvement of the carious lesion in tooth 36. She was recommended for endodontic. With mild calculus deposits and stains, she maintained a fair oral hygiene. The patient was known to be diabetic, hypertensive, and under medication, but did not follow a regular check-up schedule. During the intra-oral soft tissue examination, a round area of erythematous petechiae with a clear center of about 3 cm maximum diameter was identified on her palate (Figure 1 (Fig. 1)).

The patient was unaware of the lesion and she was asymptomatic at the time of her visit. Nevertheless, we performed a more personal anamnesis, finding that her sexual practice of fellatio, with the last instance of 3 days prior to her dental visit, was reason for the development of the palatal petechiae. Serological laboratory tests for human immunodeficiency virus (HIV) and venereal disease were negative. The etiology of her palatal lesion was explained to the patient, and she was advised to maintain good oral hygiene. 

## Discussion

Oral lesions secondary to fellatio were initially reported in the early English literature by Ronchese [6], after the earlier French report of palatal lesions by Barthelemy [7]. The characteristic clinical picture of palatal petechiae arises 2 days following the trauma and are asymptomatic. These palatal lesions usually resolve within 7 days. Fellatio-associated traumatic blood vessel injuries of the palate present as palatal ecchymoses [5], palatal erythema [8], palatal hemorrhage, palatal petechiae [9] and palatal purpura [10]. In accordance with Schlesinger et al. [9], our patient was asymptomatic and showed the signs of palatal petechiae. The clinician has to consider other etiological factors of palatal petechiae and purpura, such as blood dyscrasia, infection, trauma and tumor before reaching a final diagnosis. If the diagnosis of fellacio-associated trauma cannot be established, additional evaluations should be performed, such as complete blood cell counts with platelets and other coagulation studies to examine for blood dyscrasias, serologic examinations and cultures for Epstein-Barr virus and beta-hemolytic stretococcus infection, as well as radiologic examinations, e.g., computerized axial tomography and/or magnetic resonance imaging to rule out nasopharyngeal carcinoma [8]. Fellatio-induced submucosal hemorrhage of the palate resolves spontaneously, usually in less than a week or two. Therefore, treatment of the palatal lesion is not necessary. Recurrence of the lesions may be due to new episodes of receptive oral sex [10]. While treating the patients with oral infections secondary to fellatio, both oral and genital lesions should be resolved, as inadequately treated genital lesions are more likely the reason for the persistence of oral infection in those patients [5].

## Conclusion

A small focal area of erythema in the midline of the palate without any probable cause or any specific contributing factor are clear signs that should increase the motivation to obtain a more personal patient history. As the patients may be unaware of the etiology of the lesions, and when they hesitate to provide details of sexual history, it is important that the clinician take fellatio as a possible cause into account, based on the patient’s clinical presentation. Testing/examining the patients for sexually transmitted diseases should be considered, as these infections can be associated with fellatio.

## Notes

### Authors’ ORCID

- Karthik Shunmugavelu: 0000-0001-7562-8802
- Subhashini Paari: 0000-0001-5191-0539

### Competing interests

The authors declare that they have no competing interests.
